# Venom Proteins of the Firefly *Pyrocoelia analis* Revealed by Transcriptome Analysis

**DOI:** 10.3390/toxins18010018

**Published:** 2025-12-27

**Authors:** Guohao Liu, Chengquan Cao, Liang Chen, Rui Huang, Long Li, Er Meng, Changjun Liu, Canwei Du

**Affiliations:** 1 School of Life and Health Sciences, Hunan University of Science and Technology, Xiangtan 411201, China; 2 College of Life Sciences, Leshan Normal University, Leshan 614000, China

**Keywords:** firefly, venom, transcriptome, CRISP, phospholipase A2

## Abstract

Fireflies, which predominantly prey on various mollusks such as small snails and slugs, are renowned for their unique bioluminescence. Firefly toxins—particularly Lucibufagins (LBGs), which target the α-subunit of the sodium–potassium pump protein (ATPα)—play a crucial role in their survival strategies. However, the types and functions of venom proteins in fireflies remain to be elucidated. In this study, transcriptome sequencing was employed on the larval head of *Pyrocoelia analis* larvae, through which transcripts encoding several putative venom proteins were identified, including phospholipase A1/A2, 5′-nucleotidase, cysteine-rich secretory proteins (CRISPs), and insulin-like peptides. Structural comparison revealed that venom proteins in fireflies exhibited high sequence and structural similarity with venom proteins from various venomous animals (e.g., snakes, scorpions, spiders, and cone snails). These venom proteins may exert synergistic effects through multiple mechanisms, such as neurotoxicity, metabolic interference, and cytotoxicity, thereby playing an essential role in mollusk predation and defense against predators. Our study not only analyzes the complexity and uniqueness of *Py. analis* venom proteins but also provides a robust foundation for further exploration of the ecological adaptability and evolutionary mechanisms of these venom proteins.

## 1. Introduction

Fireflies (*Coleoptera*: *Lampyridae*) are beetles renowned for their bioluminescence. Nearly 2200 species of fireflies from 11 subfamilies have been recorded and studied [1,2]. The most prominent characteristic of this group is their unique bioluminescent trait, which has made them a focus of entomological research. They exhibit a broad ecological distribution and can be found in diverse terrestrial ecosystems, including tropical rainforests, temperate forests, wetlands, alpine meadows, and even urban environments [3]. This wide distribution reflects their remarkable ecological adaptability, which is closely linked to their complex life history traits. The life history of fireflies exhibits distinct features: the larval stage occupies most of their entire life cycle, typically lasting several months to over a year, while the adult stage is relatively short, generally persisting only a few weeks, with the primary functions of reproduction and dispersal [4,5]. Notably, most firefly larvae are obligate predators, with their prey primarily confined to the phylum Mollusca, particularly snails of the class Gastropoda [6]. In terms of predatory mechanisms, firefly larvae have evolved highly specialized morphological structures and physiological adaptations. For instance, larvae of the North American *Photinus marginellus* and the Chinese *Aquatica leii* (Lei’s aquatic firefly) possess specialized hollow mandibles, which enable efficient injection of venom into prey [6,7,8]. This injection system ensures that venom enters the prey’s body rapidly, inducing a rapid paralytic effect. More remarkably, larvae of the East Asian *Pyrocoelia atripennis* (black-winged firefly) exhibit more complex predatory behavioral traits: they can accurately track the mucus trails left by arboreal snails on the ground and vegetation via chemoreceptors, thereby locating and preying on these otherwise inaccessible prey [9,10]. This behavioral adaptation implies that the venom system of *Py. atripennis* may contain venom components with high specificity and efficiency.

Current research on the chemical defense system of fireflies primarily focuses on small-molecule toxins. Lucibufagins (LBGs)—a class of cardiotonic steroid defensive toxins—represent the most thoroughly studied firefly toxins. These compounds were initially isolated from North American *Photinus ignitus* and *Ph. marginellus* [11]. In terms of mechanism of action, LBGs specifically inhibit Na^+^/K^+^-ATPase activity, disrupting cellular ion gradients and causing cellular dysfunction and death. This mechanism is confirmed in the defensive behavior of *Lucidota atra*, which synthesizes LBGs to effectively deter predation by natural enemies such as birds and spiders [12]. Notably, certain species lacking the ability to synthesize LBGs have evolved “chemical theft” strategies; for example, female fireflies of the genus *Photuris* can mimic the flash signals of other species’ females to attract and prey on males, thereby acquiring LBGs from their bodies [13]. This cross-species transfer of chemical defense strategies even extends to vertebrates: snakes of the genus *Rhabdophis* prey on firefly larvae and store LBGs in specialized neck glands for chemical defense [14]. This phenomenon provides an excellent model for studying arms races in chemical ecology.

However, compared with the relatively well-characterized small-molecule toxins, research on firefly protein toxins is still in its early stages. In recent years, the rapid development of high-throughput sequencing technologies has provided unprecedented opportunities to study venoms from organisms that produce only trace amounts of secretions. Transcriptomic approaches, characterized by high sensitivity, high throughput, and de novo assembly capability without requiring a reference genome, have become core tools in toxin discovery research [15,16]. These methods have been successfully applied to toxicogenomic studies of various venomous animals, including snakes, spiders, and scorpions [17], significantly advancing the field of toxinology. Particularly for species like fireflies with extremely low venom yields, transcriptomics has become the most effective strategy for systematically analyzing their toxin composition. In the present study, a comprehensive transcriptomic analysis was conducted on the larval head of *Py. analis* larvae using the Illumina high-throughput sequencing platform. A high-quality transcript database was constructed and, combined with bioinformatics methods, 12 venom protein families were systematically identified. This study systematically reveals the protein compositional characteristics of firefly venom, which is not only of great significance for understanding the chemical ecology of fireflies but also provides new resources and perspectives for evolutionary research and the development of applications for animal toxins.

## 2. Results

### 2.1. Transcriptome Data Quality

**Figure 1:**
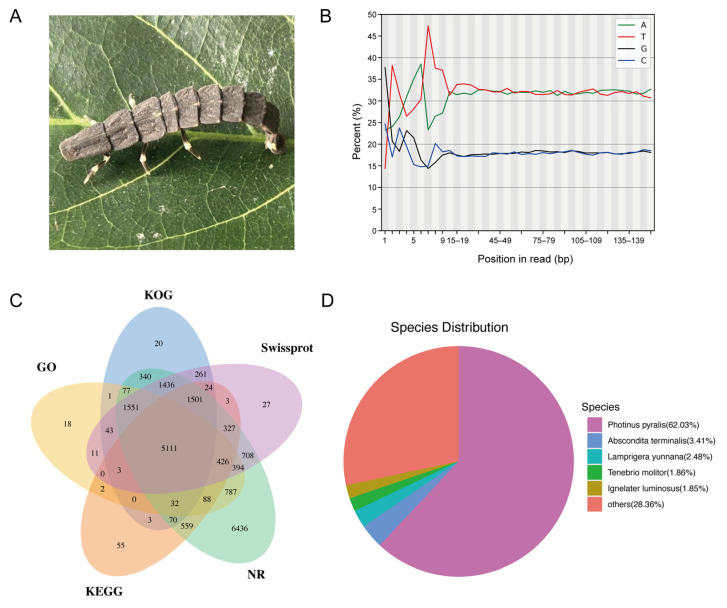
Transcriptome data analysis. (**A**) Larval *Py. analis*. (**B**) Content of the four bases: A (green), T (red), C (blue), and G (black). (**C**) Venn diagram of annotated genes across five databases: GO, KOG, SwissProt, KEGG, and NCBInr. Central overlap refers to genes annotated in all databases, while peripheral regions display single or common annotations across subsets of the databases. (**D**) Species distribution based on NCBInr annotation.

A total of 61,856,204 clean reads, equivalent to more than 9.24 billion clean nucleotides, were generated with the Illumina HiSeq^TM^ platform (MGI, Shenzhen, China). The clean data were evaluated using FastQC, and the quality metrics demonstrated very high accuracy: Q20 of 99.12% (Q20 > 95%), Q30 of 96.04% (Q30 > 90%), and GC content of 35.97% (Table 1). A total of 80,411 contigs, corresponding to over 134 million bases, were subsequently assembled from the short reads using Trinity. Subsequently, the assembled contigs were clustered and assembled into 40,892 unigenes with an average length of 1691 nucleotides. Statistical analysis of the clean data revealed that the distribution of the four nucleotide bases, as illustrated in Figure 1B, stabilized and remained balanced after the initial 10–15 base pairs. This pattern is consistent with the known characteristics of the Illumina sequencing platform.

The data were annotated using five public databases, including Eukaryotic Orthologous Group (KOG), Kyoto Encyclopedia of Genes and Genomes (KEGG), NCBI’s non-redundant protein database (NCBInr), SwissProt, and Gene Ontology (GO), and 40,892 unigenes were annotated across these databases. Among them, 10,473 unigenes were annotated in the KOG database, 8204 in KEGG, 19,843 in NCBInr, 11,826 in SwissProt, and 8544 in GO. A total of 20,314 overall annotations were obtained. Subsequently, the annotation results of the five databases were statistically analyzed, and a Venn diagram was generated to illustrate the overlap of the annotated genes among the five databases (GO, KOG, SwissProt, KEGG, and NCBInr). The central overlap refers to the common annotations of genes in all databases, while the peripheral regions indicate annotations unique to or shared among subsets of the databases (Figure 1C).

The unigene sequences were annotated via BLAST (V.2.7.1) searches against the NCBInr database, and the species distribution of the annotation results is shown in Figure 1D. The top BLAST hits indicated that 62.03% of the unigenes matched the North American firefly *Photinus pyralis*. In contrast, 3.41% and 2.48% of the unigenes matched *Abscondita terminalis* and *Lamprigera yunnana*, respectively. In addition, a small proportion of unigenes showed top matches to non-firefly species, such as *Tenebrio molitor* (1.86%) and *Ignelater luminosus* (1.85%).

### 2.2. Excavation of Venom Proteins

To mine venom proteins from firefly larval heads, transcriptomic data were functionally annotated using five major public databases (KOG, KEGG, NCBInr, SwissProt, and GO), yielding relatively comprehensive results. Preliminary analysis of the annotation results suggested that fireflies possessed venom proteins shared with other venomous taxa, such as snakes, scorpions, and bees. To systematically screen for such proteins, the standardized “venom protein family” classification terms provided by the VenomZone (https://venomzone.expasy.org/) resource were used—a web platform that curates and organizes toxin data across six major venomous taxa (snakes, scorpions, spiders, cone snails, anemones, and insects) and provides comprehensive information on venom protein families categorized by taxonomy, activity, and family of toxin proteins. By applying these curated family terms to the transcriptome annotation results, 19 transcript sequences were initially flagged as potential venom protein candidates. However, VenomZone alone was insufficient for a comprehensive analysis. To expand and rigorously validate the toxin dataset, we implemented a multi-step filtering strategy. First, the functional annotation results from all five databases (KOG, KEGG, NCBInr, SwissProt, and GO) were manually reviewed to identify transcripts with putative toxin-related annotations. Subsequently, the corresponding amino acid sequences of these candidate transcripts were extracted and subjected to targeted BLASTp homology analysis (E-value threshold of 1 × 10^−5^ and minimum sequence identity of 50%) against the NCBI non-redundant (nr) protein database. Only sequences demonstrating clear and significant homology (meeting the above thresholds) to previously characterized venom proteins were retained for further analysis. Finally, a total of 12 venom protein families comprising 99 transcripts were identified, each transcript corresponding to a predicted amino acid sequence (Table 2).

### 2.3. Venom Protein Family Analysis

Based on the results of BLASTp, six venom protein families widely studied in other venomous taxa were selected for detailed analysis, including cysteine-rich secretory proteins (CRISPs), insulin, phospholipase A2 (PLA2), scoloptoxin SSD14, phospholipase A1 (PLA1), and 5′-nucleotidase. For each family, one representative transcript with a complete open reading frame (ORF) was randomly chosen and named CRISP2, PLA2PA4, SSD14L2, PLA1, and 5-NT.

#### 2.3.1. PLA1

**Figure 2:**
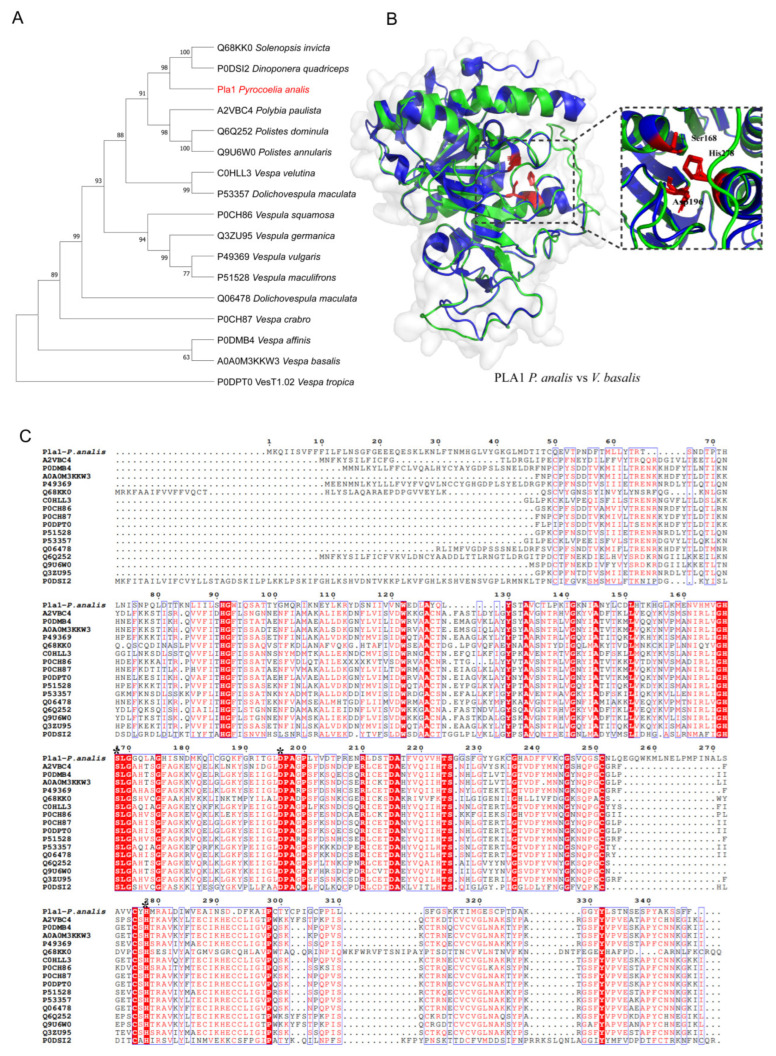
Evolutionary and structural analysis of PLA1 from *Py. analis.* (**A**) The phylogenetic tree was constructed from protein sequences retrieved from UniProt databases (represented by their respective database accession numbers) using the maximum likelihood method with the Jones–Taylor–Thornton (JTT) amino acid substitution model. Branch support values were evaluated via 1000 bootstrap replicates, and bootstrap values for key nodes are labeled. The monophyletic group containing *Py. analis* PLA1 is highlighted in red on the corresponding branch. (**B**) The predicted 3D structure of *Py. analis* (**A**) PLA1 (green) was superimposed with the template protein V. basalis PLA1 (PDB: 4QNN, blue). Residues of the catalytic triad (corresponding to Ser168, Asp196, and His278 in *Py. analis*) were displayed as stick models, demonstrating high structural conservation of the active site. (**C**) Sequence alignment of PLA1 homologs, the highly conserved catalytic triad (Ser-His-Asp) in wasp venom PLA1s, corresponded to residues Ser168, Asp196, and His278 in *Py. analis* PLA1. These key residues are marked with asterisks (*) in the sequence alignment.

To clarify the evolutionary origin and function of the venom protein family PLA1 in fireflies, three venom PLA1 protein transcripts were identified in the larval head of *Py. analis* through transcriptomic analysis (Table 2), and one representative sequence (PLA1) was selected for phylogenetic analysis. First, PLA1 homologous sequences were obtained through a comprehensive search of UniProt (https://www.uniprot.org/), followed by validation and expansion using BLASTp. After removing redundant sequences, a set of representative sequences covering different insect taxa was acquired. A phylogenetic tree was constructed using the maximum likelihood method in MEGA v11.0.13 (Figure 2A). This tree revealed that *Py. analis* PLA1 shared a close evolutionary relationship with PLA1s from Formicidae (e.g., *Dinoponera quadriceps* and *Solenopsis invicta*) and *Vespidae* (e.g., *Vespa velutina* and *Vespula germanica*). This result was highly consistent with previous reports that venom PLA1s of social hymenopteran insects exhibited extreme evolutionary conservation [18]. Sequence alignment results showed that the highly conserved catalytic triad (Ser-His-Asp) in wasp venom PLA1s corresponds to residues Ser168, Asp196, and His278 in *Py. analis* PLA1 [19] (Figure 2C). Homology modeling based on the wasp PLA1 structure (*Vespa basalis*, PDB: 4QNN) further confirmed that these key residues form a complete active site in the three-dimensional structure of *Py. analis* PLA1 [19] (Figure 2B). Previous studies indicated that wasp venom PLA1s could hydrolyze the ester bond at the sn-1 position of cell membrane phospholipids, leading to membrane disruption and cytolysis; they also acted as potent platelet activators, inducing thrombosis [20]. Based on the aforementioned evolutionary association and structural conservation, we hypothesized that during predation, *Py. analis* PLA1 may synergize with other toxins through similar membrane-disrupting and procoagulant mechanisms, collectively inducing rapid paralysis and tissue degradation of prey [21].

#### 2.3.2. PLA2

**Figure 3:**
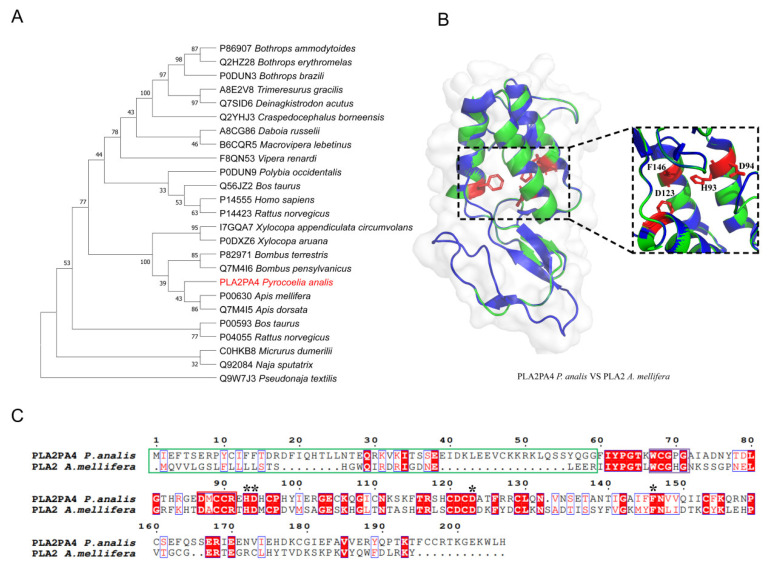
Evolutionary and structural analysis of PLA2 from *Py. analis.* (**A**) Maximum likelihood phylogenetic tree of PLA2 homologs. The tree was constructed using the JTT amino acid substitution model with 1000 bootstrap replicates; support values for key nodes are shown. The tree illustrates the evolutionary relationship between *Py. analis* PLA2PA4 (marked in red) and PLA2s from Apidae species (e.g., *A. mellifera* and *A. dorsata*). (**B**) Structural superimposition of predicted *Py. analis* PLA2PA4 and the template protein. The predicted 3D structure of *Py. analis* PLA2PA4 (green) is superimposed with the template protein *A. mellifera* PLA2 (PDB: 1POC, blue). Key residues of the catalytic network (His93, Asp94, Phe146, and Asp123) are displayed as stick models, demonstrating structural conservation of the active site. (**C**) Sequence alignment of PLA2. Homologs’ putative signal/propeptide regions, conserved Ca^2+^-binding sites, and catalytic network residues are marked with green, purple, and red boxes, respectively. The alignment shows high consistency in key functional sites between *Py. analis* PLA2PA4 and bee venom PLA2 (UniProt: P00630). These key residues are marked with asterisks (*) in the sequence alignment.

Seven PLA2 transcripts were identified in *Py. analis* via transcriptomic analysis (Table 2), and one representative sequence (PLA2PA4) was selected for phylogenetic analysis. Phylogenetic reconstruction (Figure 3A) showed that *Py. analis* PLA2PA4 shared a close evolutionary relationship with species of the Apidae family, such as *Apis mellifera* (honeybee) and *Apis dorsata* (giant honeybee). Sequence and structural alignment analyses indicated that PLA2PA4 contained a putative signal peptide/propeptide region, and its conserved Ca^2+^-binding site and catalytic network residues (His93, Asp94, Phe146, and Asp123) were highly similar to those of *A. mellifera* bee venom PLA2 (UniProt: P00630) [22] (Figure 3C). Of particular note, the positions of key cysteine residues involved in disulfide bond formation in PLA2PA4 corresponded exactly to those in bee venom PLA2—a feature crucial for maintaining structural stability and catalytic function [23]. Three-dimensional structural modeling based on *A. mellifera* PLA2 (PDB: 1POC) further confirmed that PLA2PA4 possessed a complete catalytic active site and a typical PLA2 fold conformation (Figure 3B). Studies have demonstrated that bee venom PLA2 (Api m 1) is not only a major allergen that induces strong immune responses but also exhibits direct toxic effects [24], including hemolytic activity and the promotion of inflammatory responses [25,26]. Given the high conservation of PLA2PA4 with bee venom PLA2 across evolution, sequence, and structure, we hypothesized that *Py. analis* PLA2PA4 may disrupt the tissue integrity of prey through a similar membrane lipid hydrolysis mechanism. This finding not only reveals the important role of PLA2 in the firefly venom system, but also offers a new perspective for understanding the functional evolution of this toxin family in arthropods.

#### 2.3.3. Insulin

**Figure 4:**
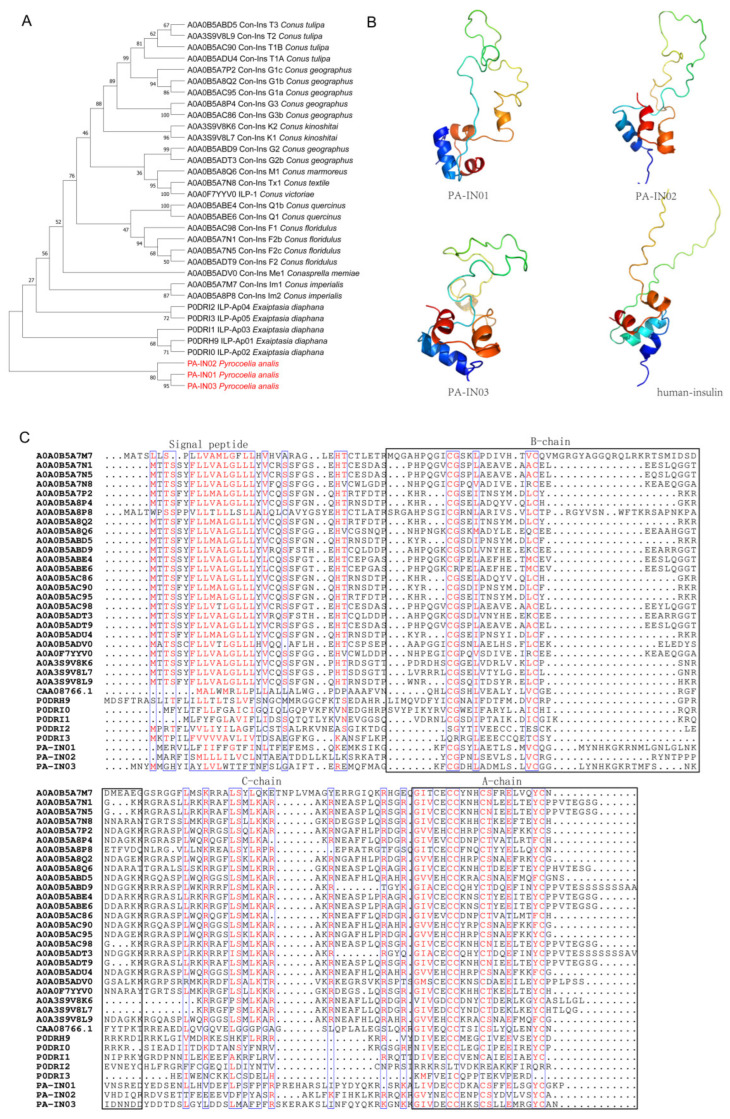
Evolutionary and structural analysis of insulin from *Py. analis*. (**A**) Maximum 1000. bootstrap replicates; support values for key nodes are shown. *Py. analis* insulin PA-IN01-03 (marked in red) form a phylogenetically significant clade together with insulin sequences from *Conus* species and *E. diaphana*. (**B**) Comparison of predicted 3D structures of three Py. analis insulin transcripts. Predicted 3D structures of the three *Py. analis* insulin transcripts (PA-IN01-03) were obtained via homology modeling, using human insulin (PDB: 6PXV) as the 3D structure template. Structures of the different transcripts (B chain, C chain, and A chain) are labeled in different colors, highlighting their conservation and divergence in spatial conformation. (**C**) Sequence alignment of insulin peptides. Conserved sites in the signal peptide region, B chain, C chain, and A chain are marked with black boxes, demonstrating the sequence similarity between *Py. analis* insulin and known venom insulin in key functional domains.

Three insulin-like peptide (PA-IN01-3) transcripts were identified in *Py. analis* via transcriptomic analysis (Table 2). Phylogenetic analysis (Figure 4A) revealed that although these transcripts formed an independent clade in the phylogenetic tree, they still exhibited an evolutionary affinity with venom insulins from species such as *Exaiptasia diaphana* (sea anemone) and several *Conus* species. This suggests that these peptides may function as toxins to support predatory strategies rather than perform conventional blood glucose regulation. Venom insulin from cone snails has been shown to rapidly induce a hypoglycemic coma in prey to facilitate predation [23]. Further sequence alignment (Figure 4C) demonstrated that *Py. analis* insulin was highly conserved in key functional regions, including the signal peptide, B chain, C chain, and A chain. Notably, in the A chain and B chain domains involved in receptor binding, the residue composition of *Py. analis* insulin exhibited significant similarity to that of known venom insulins. This characteristic was consistent with previous reports: in the insulin venom sequences of humans, *Conus* species, and *E. diaphana*, the A chain of insulin follows the C-C-C-C cysteine pattern, while the B chain has a C-C pattern [27]. These cysteine residues form three characteristic disulfide bonds, reflecting the evolutionary conservation of this structural motif within the insulin superfamily. In addition, 3D structural modeling (Figure 4B) showed that *Py. analis* insulins shared high spatial conformational consistency with cone snail venom insulins, especially in key regions that maintain insulin folding and mediate receptor interaction. Conservation of sequence and structure strongly suggested that hypoglycemic coma in prey may be induced by *Py. analis* insulins through a similar mechanism, thereby aiding predation. This study not only provides a new case for the evolution of “weaponization” of animal hormones, but also offers a potential molecular template for the development of novel hypoglycemic drugs based on insect toxins.

#### 2.3.4. CRISP

**Figure 5:**
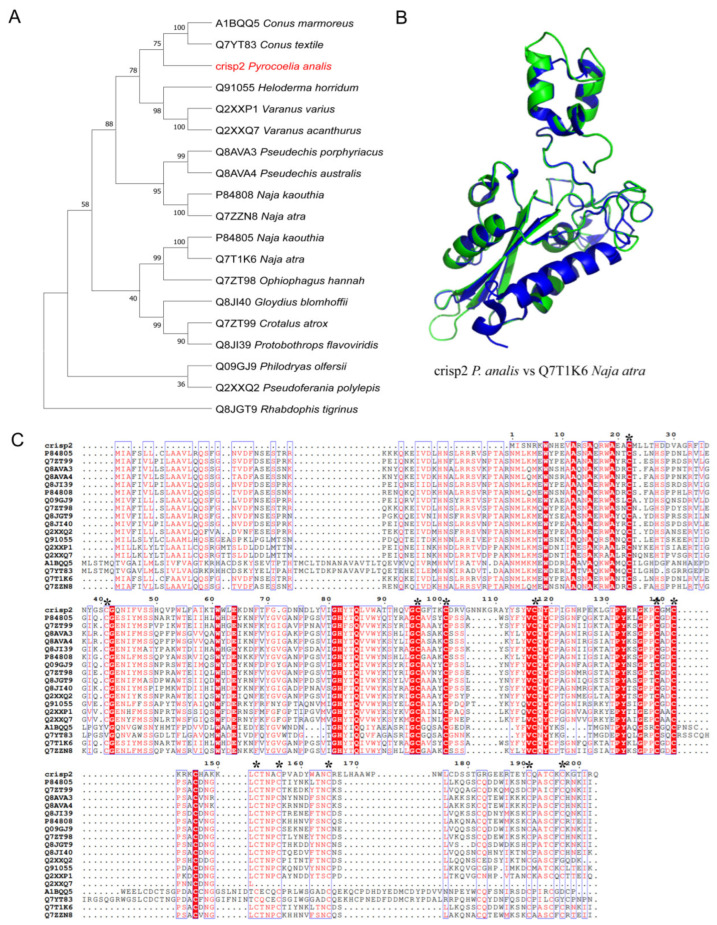
Evolutionary and structural analysis of the CRISP toxin from *Py. analis*. (**A**) Phylogenetic analysis was performed with 1000 bootstrap replicates. support values for key nodes are shown. *Py. analis* CRISP2 (marked in red) forms a phylogenetically significant clade together with CRISP/CRVP proteins from *Conus* (*cone snail*), *Varanus* (*monitor lizard*), and *Viperidae* species. Bootstrap values for key nodes are labeled. (**B**) Structural superimposition of predicted *Py. analis* CRISP2 and snake venom CRVP protein Natrin. The predicted three-dimensional structure of *Py. analis* CRISP2 (green) is superimposed with that of the snake venom CRVP protein Natrin (PDB: 3MZ8, blue). Conserved structural folding patterns and functionally relevant cysteine residues are displayed as stick models. (**C**) Sequence alignment of CRISP homologs. Sequence alignment of CRISP homologs revealed high conservation of *Py. analis* CRISP2 with snake venom CRVPs at key functional sites. These key residues are marked with asterisks (*) in the sequence alignment.

To clarify the evolutionary origin and function of CRISP toxins in fireflies, one CRISP toxin transcript was identified in the larval head of *Py. analis* (black-winged firefly) via transcriptomic analysis (Table 2) and named CRISP2. Phylogenetic analysis (Figure 5A) showed that *Py. analis* CRISP2 formed an evolutionary clade with cone snail species (e.g., *Conus marmoreus* and *Conus textile*) and monitor lizard species (e.g., *Varanus varius* and *Varanus acanthurus*), and exhibited a close evolutionary affinity with elapid snake species (e.g., *Naja kaouthia*, *Naja atra*, and *Ophiophagus hannah*). Further sequence alignment analysis (Figure 5C) revealed that CRISP2 was highly conserved with snake venom CRVPs at key sites of the N-terminal signal peptide and CAP domain [28], particularly showing significant similarity in the spatial conformation of cysteine residues associated with ion channel interaction [29]. Through 3D structural modeling, high overall folding similarity was observed between CRISP2 and snake venom CRVP (Natrin, PDB ID: 3MZ8), further supporting the conservation of its functional structure (Figure 5B). Studies have demonstrated that CRISPs in snake venom exert neuromuscular toxicity through multiple mechanisms, including inhibiting cyclic nucleotide-gated ion channels [30], ryanodine receptors [31,32], and blocking potassium channels [33]. Based on the aforementioned conservation in evolution, sequence, and structure, we suggest that firefly CRISP2 may represent a functional homolog of known venom CRISPs. This finding provides new molecular evidence for understanding the convergent evolution of functions of the CRISP superfamily in the venom systems of invertebrates and vertebrates.

#### 2.3.5. 5′-Nucleotidase

**Figure 6:**
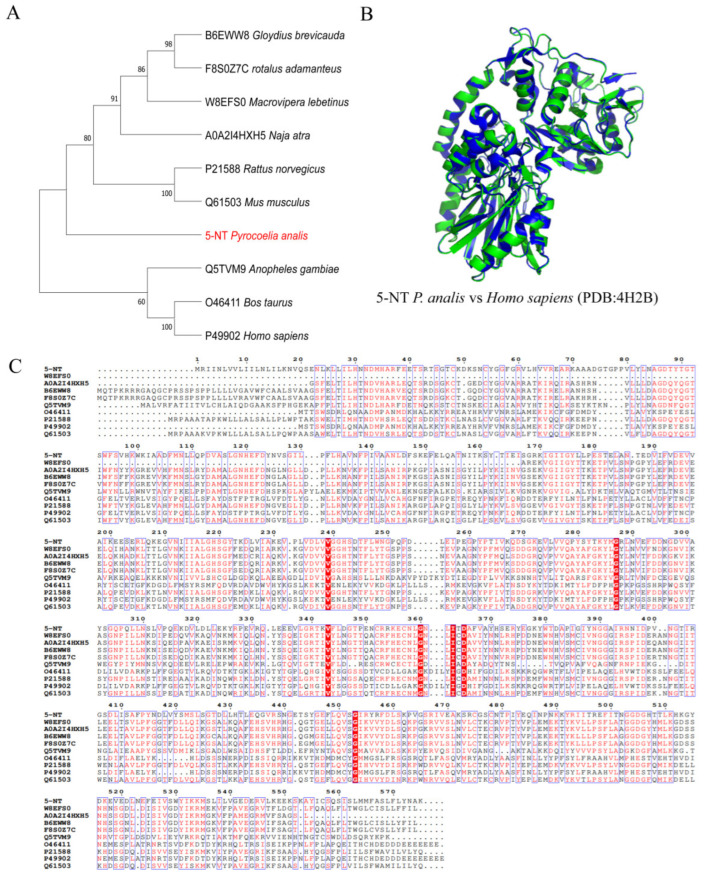
Evolutionary and structural analysis of 5′-nucleotidase from *Py. analis.* (**A**) Phylogenetic analysis was performed with 1000 bootstrap replicates, support values for key nodes are shown. *Py. analis* 5-NT (marked in red) forms a well-supported clade with 5′-nucleotidases from elapid species (*N. atra*, *M. lebetinus*) while maintaining a close evolutionary distance from species of *Muridae* and Culicidae. (**B**) The predicted 3D structure of *Py. analis* 5-NT (green) is superimposed with that of the template 5′-nucleotidase (PDB: 4H2B, blue). (**C**) Sequence alignment of 5′-nucleotidase homologs. The sequence alignment of 5′-nucleotidase homologs showed high conservation of *Py. analis* 5-NT with snake venom homologs at functional sites.

Analysis of 5′-nucleotidase revealed that four transcripts of this family were identified in the larval head transcriptome of *Py. analis* (Table 2), and one representative sequence (5-NT) was selected for phylogenetic analysis (Figure 6A). Phylogenetic reconstruction indicated that the representative 5′-nucleotidase sequence (5-NT) of *Py. analis* showed close phylogenetic affinity to a diverse clade comprising mammals (*Rattus norvegicus* and *Mus musculus*), viperids (*Macrovipera lebetina*), and elapids (*Naja atra*). Sequence alignment demonstrated that key amino acid residues in the enzyme active center and substrate-binding pocket of 5-NT were highly conserved with those of snake venom homologs (Figure 6C). Three-dimensional structural modeling based on 5′-nucleotidase (PDB: 4H2B) further confirmed the integrity of its catalytic domain (Figure 6B). 5′-nucleotidases in snake venom have been shown to effectively inhibit platelet aggregation by hydrolyzing AMP to produce adenosine and synergistically exert potent anticoagulant effects with other toxins (e.g., ADPase, phospholipase, and disintegrin) [34]. Based on the aforementioned evolutionary relationships and structural conservation, it is plausible that *Py. analis* 5-NT may interfere with nucleotide-related physiological processes in prey, although its specific effects on the distinct circulatory and coagulation systems of mollusks remain to be elucidated.

#### 2.3.6. Scoloptoxin SSD14

**Figure 7:**
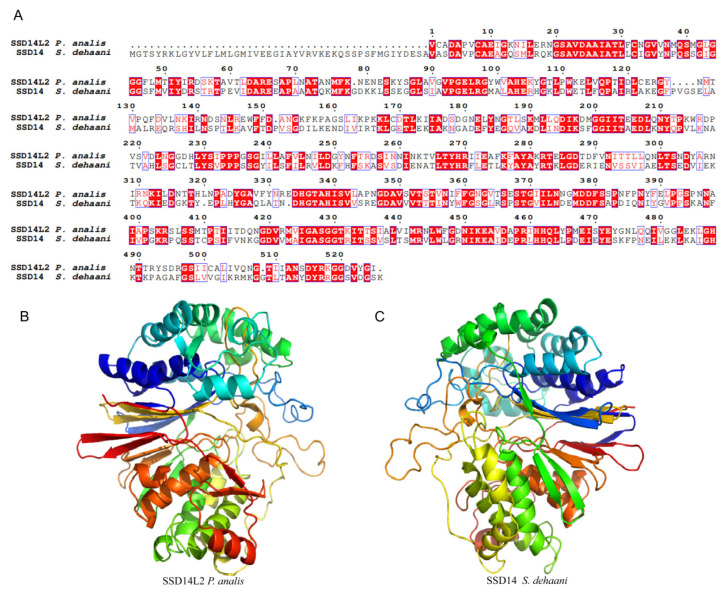
Sequence and structural analysis of Scoloptoxin SSD14 from *Py. analis.* (**A**) Sequence alignment of *Py. analis* SSD14L2 and centipede venom Scoloptoxin SSD14. (**B**,**C**) A comparison of predicted 3D structures of *Py. analis* SSD14L2 and centipede scoloptoxin SSD14. Both structures were generated via homology modeling using PDB: 4y23 as the template.

In the transcriptome of the *Py. analis* larval head, three transcripts belonging to the Scoloptoxin SSD14 family were identified, with detailed information presented in Table 2. Since SSD14-like toxins were previously only reported in centipede venoms, this study focused on sequence and structural analyses to elucidate their potential functions in fireflies. For sequence alignment, SSD14L2 was selected as a representative transcript for detailed characterization, and the results (Figure 7A) showed that the *Py. analis* SSD14L2 transcript exhibited significant sequence similarity to centipede Scoloptoxin SSD14 in multiple key regions, particularly around the putative enzymatic active site [35]. Further 3D structural modeling demonstrated that SSD14L2 shared a highly conserved overall fold with its centipede homolog (Figure 7B). Previous studies indicated that SSD14 in centipede venoms could interfere with glutathione metabolism by mimicking the activity of γ-glutamyl transferase (GGT) and triggering platelet aggregation [35,36]. Based on the conservation of sequence and structure, it was hypothesized that *Py. analis* SSD14L2 may disrupt the redox balance and coagulation system of prey through a similar mechanism, thereby synergizing with other toxins during predation to enhance the overall toxicity of the venom.

### 2.4. Expression Level Validation

**Figure 8:**
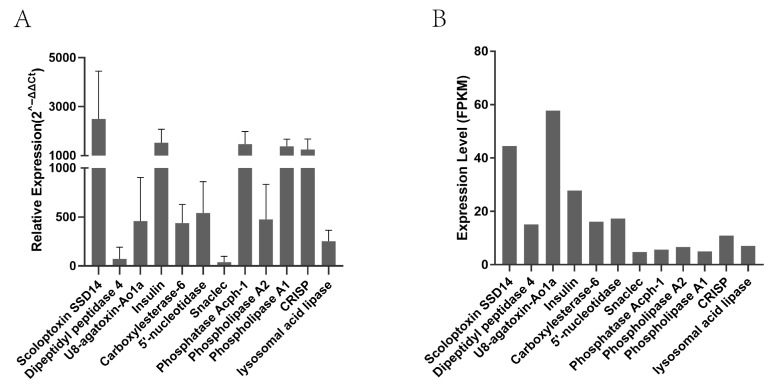
(**A**) Expression levels determined using qPCR analysis (*n* = 4). (**B**) Expression levels determined using transcriptome data.

The expression levels of putative toxin proteins in the *Py. analis* larval head transcriptome were analyzed using the FPKM method, and the results showed significant differences in the expression levels of different toxin genes (Figure 8B). To further verify the expression patterns of toxin genes, the expression levels of representative toxin genes were detected via quantitative real-time PCR (qPCR) (*n* = 4) (Figure 8A). The qPCR results indicated that the expression levels of scorpiontoxin SSD14, phospholipase A1, CRISP, and insulin-like toxin were relatively high, while the expression levels of dipeptidyl peptidase 4 and Snaclec were relatively low. A comprehensive comparison of results from the two analytical methods revealed that most toxin genes exhibited consistent expression trends between transcriptome sequencing and qPCR. Among these, scorpiontoxin SSD14 and insulin-like toxin showed significant and consistent expression characteristics in both methods.

## 3. Discussion

As a model organism for bioluminescence research, the venom system of fireflies has long been overlooked. In recent years, with the advancement of toxicogenomics technologies, a growing body of studies has demonstrated that firefly larvae use their venom system to prey on mollusks [6,7,8], while adult fireflies employ toxins for chemical defense [12]. In this study, the composition of toxin proteins in *Py. analis* was systematically identified for the first time through transcriptome analysis, providing a new perspective for understanding the ecological adaptability and evolutionary mechanisms of fireflies. More importantly, the unique and diverse properties of firefly toxins offer valuable resources for the development of novel drugs.

In this study, firefly venom was found to contain 12 categories of venom proteins, including enzymatic toxins (phospholipases and nucleotidases) and non-enzymatic toxins (CRISPs and insulin-like peptides). This diversity reflected the multifunctionality of the firefly venom system: it was used for predation, such as through the neurotoxicity of PLA2 and metabolic disruption by insulin-like peptides [37], and also for defense, such as via the allergenicity of PLA1 and ion channel regulation by CRISPs [38]. Compared with other venomous animals, firefly toxins exhibited a unique combination pattern. They not only retained typical arthropod venom characteristics, such as phospholipases, but also evolved functional components similar to those in higher animal venoms, such as insulin-like toxins. The structural similarity between firefly venom peptides and those from venomous animals, such as snakes, scorpions, spiders, and cone snails, indicates significant convergent evolution; for example, CRISPs function as ion channel regulators in both snake and firefly venoms [39]. Insulin-like toxins had independently evolved the ability to disrupt metabolism in cone snails and fireflies [27]. Phospholipases exerted toxicity through membrane lipid hydrolysis in snakes, scorpions, and fireflies alike [25,40,41]. This evolutionary pattern, in which functions converge while structures diverge, provided an ideal model for studying evolutionary innovation in animal venom.

### 3.1. Comparison with Arthropod Venom

Compared with spider toxins, firefly toxins lacked diverse neuropeptide components but retained key enzymatic toxins, such as phospholipases. Unlike scorpion venom, which primarily exerts its toxicity through neuroparalysis, firefly toxins primarily target metabolic disruption. This characteristic was likely closely related to their ecological strategy of obligate predation on mollusks [6]. When compared with venoms of hymenopteran insects (e.g., bees and wasps), firefly venom contained more abundant digestive enzymes, an adaptation to its feeding strategy of external digestion. Notably, this study identified PLA1—a characteristic component of wasp venom—in firefly venom, though it may exert functions through different molecular mechanisms [21]. Phylogenetic analysis (Figure 2A) showed that firefly PLA1 clusters with PLA1 from wasps (e.g., *Polybia paulista* and *Polistes dominula*) and ants (e.g., *Solenopsis invicta*) into a monophyletic group with high support values, suggesting they may originate from a common ancestor. However, firefly PLA1 formed an independent clade in the phylogenetic tree, implying it may have undergone functional specialization during adaptation to the obligate predation of mollusks. Structural analysis showed that firefly PLA1 retained a typical catalytic triad (Ser168, Asp196, and His278) [21] and a complete substrate-binding pocket. These structural features were highly conserved with wasp PLA1 (Figure 2B,C). Literature studies indicated that wasp PLA1, as a major allergen, could trigger strong immune responses by hydrolyzing cell membrane phospholipids. It also exhibited hemolytic activity and the ability to induce platelet aggregation [29,31]. Although firefly PLA1 had a similar structural basis, its function in the venom system may focus more on tissue decomposition during predation rather than defensive allergic reactions. This functional divergence may stem from differences in substrate selectivity or target specificity: wasp PLA1 primarily acted on the immune and circulatory systems of vertebrates [21], while firefly PLA1 may specifically target the tissues and coagulation systems of mollusks. This study identified PLA1 toxin in firefly venom for the first time. The evolutionary conservation and structural similarity of firefly PLA1 provide a new perspective on the functional evolution of enzymatic toxins in animal venoms. The specific mechanism of action of firefly PLA1 in predation—including whether it retained hemolytic or procoagulant activity, and how it synergized with other toxins—requires further experimental verification.

### 3.2. Comparison with Vertebrate Venom

Compared with snake venom, firefly venom exhibited lower complexity but retained key functional modules: the anticoagulant system (5′-nucleotidase) [42], the neurotoxic system (PLA2) [23], and the cytotoxic system (CRISPs) [43]. This “simplified yet complete” venom system provided an ideal model for investigating the minimal essential components required for toxin function. Structural alignment revealed that firefly PLA2 retained a catalytic network architecture and disulfide bond pairing pattern similar to those of snake venom PLA2, suggesting significant functional convergent evolution. Specifically, the spatial conformation of catalytic residues (His93, Asp94, Phe145, and Asp123) in firefly PLA2 was highly conserved with that in snake venom PLA2, which provided a structural basis for their similar enzymatic activity and neurotoxicity. Functional studies indicated that snake venom PLA2 (e.g., β-bungarotoxin) induced respiratory paralysis via presynaptic neurotoxicity while also exhibiting hemolytic activity and myotoxicity [25,44,45]. Although firefly PLA2 may exert its effects through a similar membrane lipid hydrolysis mechanism, significant differences existed in its target and ecological function: snake venom PLA2 primarily acted on the neuromuscular system of vertebrates [45], whereas firefly PLA2 may specifically target the nervous system of mollusks. Notably, the evolutionary independence of PLA2 between fireflies and snakes stands in sharp contrast to their structural and functional similarity. This contrast provided a typical case for studying the functional convergent evolution of toxins. The “simplified” characteristics of firefly PLA2 made it an ideal model for dissecting the molecular mechanism underlying PLA2 neurotoxicity.

### 3.3. Discovery of Rare Toxins and Their Evolutionary Significance

This study identified several toxin components rarely reported in insect venoms within *Py. analis* venom, significantly expanding current understanding of the diversity and evolutionary potential of insect venom systems. First, insulin-like toxins were identified in fireflies. Such toxins had previously been reported only in mollusks, such as cone snails [27], where their functions were primarily associated with inducing hypoglycemic shock in prey to aid predation. The results of this study suggest that insulin, as a metabolic regulatory tool, may have a broader distribution and independent evolutionary history in animal venom systems. Of particular note was the discovery of SSD14-like toxins. This toxin family was previously primarily found in centipede venom, where they interfered with glutathione metabolism and induced platelet aggregation by mimicking γ-glutamyl transferase activity [35]. Their presence and potential role in firefly venom contribute to understanding the functional diversification of this enzyme family within animal venoms. Additionally, the Snaclec domain [46] identified in the venom—typically recognized as a characteristic component of snake venom C-type lectin-like proteins—was rarely found in insects. In snake venom, such components mostly exert procoagulant or anticoagulant effects by interfering with platelet function and coagulation cascades. Their presence in firefly venom suggested that certain toxin structural modules may be independently recruited and functionally integrated across different animal groups through mechanisms such as horizontal gene transfer or deep homology. As specialized mollusk predators, fireflies’ venom systems provided a unique and valuable window for understanding the origin, migration, and functional innovation of animal toxins.

### 3.4. Pharmaceutical Development Potential

The unique structural and functional properties of animal venom proteins offer abundant opportunities for their application in pharmaceutical development. First, as ion channel modulators, CRISP toxins have been shown in snake venom and scorpion venom studies to regulate cyclic nucleotide-gated ion channels and ryanodine receptors [47]. The high conservation of firefly CRISP2 with snake venom CRVPs in terms of structure and key functional sites suggests it has the potential to be developed into novel analgesics or antiarrhythmic drugs; its ability to act on specific ion channels may help to reduce the side effects of existing drugs. Second, insulin-like toxins exhibited a unique mechanism of action that was independent of vertebrate insulin receptors. The evolutionary relationship between firefly insulin and cone snail venom insulin, as well as the specificity of firefly insulin in key functional domains [27], provided a valuable structural template for developing novel drugs for diabetes treatment—especially for designing insulin analogs with lower hypoglycemia risk. Third, although PLA2 toxins were conserved with bee venom PLA2 in catalytic networks and calcium ion-binding sites [21], there may be significant differences in their substrate specificity and target of action. Such differences endow them with unique value in the development of anti-inflammatory drugs, particularly for targeting specific phospholipid metabolic pathways in inflammatory diseases. In addition, 5′-nucleotidases have been proven to possess potent anticoagulant activity in snake venom studies [48]. The high similarity of firefly 5′-nucleotidase to its snake venom homologs in the catalytic domain suggests it may serve as a lead compound for novel antithrombotic drugs, and its unique selectivity may enable better control of bleeding risk. It should be emphasized that although these toxin components showed promising application prospects, their actual translation in pharmaceutical development still requires systematic toxicological evaluation and structural optimization. As a relatively unexplored pharmaceutical resource pool, firefly toxins deserve more in-depth functional verification and mechanism research.

### 3.5. Limitations of the Present Study

Although the present study systematically revealed the compositional characteristics of *Py. analis* venom through transcriptomic analysis, several research limitations warrant attention. First, the toxin identification results based on transcriptomic data still require verification via proteomic approaches. The abundance of transcripts did not necessarily fully reflect the actual expression and secretion levels of venom proteins; subsequent studies should directly identify the venom’s protein composition using techniques such as mass spectrometry. Second, the inference of toxin functions in this study relied mainly on sequence homology and structural conservation analyses, which lacked experimental evidence. To address this, we initiated the heterologous expression of *Py. analis* PLA1 and PLA2 toxins, aiming to verify their enzymatic activity, substrate specificity, and potential toxic effects through in vitro functional experiments. Furthermore, the physiological functions and ecological significance of these toxins under natural conditions remain to be further explored. Future studies should integrate in vivo experiments to analyze the specific roles of individual toxins in predatory or defensive behaviors, as well as their association with fireflies’ ecological adaptability.

## 4. Conclusions

This study is the first to systematically identify the toxin composition of the firefly *Py. analis*, revealing the previously unrecognized complexity and uniqueness of its venom system. Compared with toxins from other venomous animals, a significant case of convergent evolution was identified. Firefly toxins not only have crucial ecological significance, but also represent valuable resources for drug development. Future research should focus on verifying their functions and elucidating their mechanisms of action to fully exploit their scientific and application value.

## 5. Materials and Methods

### 5.1. RNA-Seq

Ten larvae of *Py. analis* were collected from Tunchang County, Hainan Province, China. Sequencing was conducted at Wuhan Grandomics Biotechnology Co., Ltd., using the Illumina HiSeq^TM^ platform. Total RNA from larval head tissues was extracted via homogenization on dry ice with TRIzol reagent (TIANGEN, Beijing, China), according to the manufacturer’s instructions. Polyadenylated RNA was enriched from total RNA using Dynabeads mRNA Purification Kit (Invitrogen, Carlsbad, CA, USA). First-strand cDNA was synthesized using random primers and reverse transcriptase, followed by second-strand synthesis. The generated double-stranded cDNA was repaired, adenylated at the 3′-end, and ligated to the sequencing adapter according to the manufacturer’s library construction protocol. The fragments bound to the adapter were amplified via PCR and purified using MGIEasy DNA Clean Beads (MGI, Shenzhen, China). The quality of the library was assessed using an Agilent 2100 Bioanalyzer (Agilent Technologies, Santa Clara, CA, USA).

Double-stranded PCR products were heat-denatured and circularized using a splint oligonucleotide provided in the MGIEasy Circularization Module (MGI, Shenzhen, China). The resulting single-stranded circular DNA (ssCir DNA) was used as the final sequencing library. The qualified libraries were sequenced on the DNBSEQ-T7RS platform (MGI, Shenzhen, China).

### 5.2. Bioinformatics Analysis

Transcriptome assembly and annotation analyses were performed by Grandomics Biotechnology Co., Ltd. (Wuhan, China). Raw sequencing reads were validated using Fastp (https://github.com/OpenGene/fastp (accessed on 8 February 2024)). The clean reads were then evaluated using FastQC (http://www.bioinformatics.babraham.ac.uk/projects/fastqc (accessed on 15 February 2024)) to determine key quality indicators, such as Q20, Q30, and GC content. Transcriptome assembly was performed using the Trinity program (v2.9.0). To reduce redundancy, clean reads were aligned to the assembled transcripts, and transcripts with zero coverage were excluded. The most similar sequences were clustered to obtain the final set of unigenes. Functional annotation of unigenes was performed using several public databases, including KOG, KEGG, NCBInr, SwissProt, and GO, to predict their potential biological functions. The expression levels of unigenes were calculated using the FPKM method at Grandomics Biotechnology Co., Ltd.

### 5.3. Sequence Alignment and Homologous Modeling

The amino acid sequences of candidate toxin proteins were derived from *Py. analis* larval head transcriptome data (Table 3). Homologous sequences were primarily retrieved from the UniProtKB database. Searches were conducted using keywords relevant to each toxin family (e.g., “phospholipase A1”, “CRISP”, and “venom”). Sequences were selected to cover a broad taxonomic distribution across venomous and non-venomous animals, with a focus on well-annotated, full-length homologs. The initial selection was further validated and expanded through BLASTp searches against the NCBI non-redundant (nr) protein database (E-value threshold of 1 × 10^−5^; minimum sequence identity of 50%). Redundant sequences from the same species or highly similar isoforms were excluded to maintain a balanced dataset. After removing duplicate sequences, the remaining homologous proteins were used as targets for sequence alignment. Multiple sequence alignment was performed using the MUSCLE algorithm in the MEGA software (v11.0.13) with default parameter settings. The alignment results were visualized using the ESPript 3.0 online tool (https://espript.ibcp.fr/ESPript/ESPript/index.php (accessed on 18 November 2024)). Three-dimensional protein structure prediction was performed using the SWISS-MODEL online server (https://swissmodel.expasy.org/ (accessed on 15 November 2024)). Template proteins were sourced from the PDB database (https://www.rcsb.org/ (accessed on 15 November 2024)). Alternatively, crystal structures with the highest homology to the target sequence and experimentally verified were selected as templates. All predicted structural models were subjected to structural superimposition and visual analysis using the PyMOL Molecular Graphics System (v3.1.1). Protein structure alignment was completed using the “align” command built into PyMOL.

### 5.4. Phylogenetic Tree Construction

Phylogenetic trees were constructed using the Maximum Likelihood method in MEGA (v11.0.13). The JTT model was employed as the substitution model for protein sequences. The reliability of branches was evaluated using the Bootstrap method with 1000 replicate samplings. The resulting phylogenetic trees are presented without specifying a root. The generated phylogenetic tree files were exported, graphically modified, and typeset using Adobe Illustrator (v2023).

### 5.5. qPCR

Total RNA was extracted from the heads of twelve larvae using Trizol reagent (Beyotime, Shanghai, China) and reverse-transcribed into cDNA using HiScript III RT SuperMix (Vazyme, Nanjing, China). The synthesized cDNA was used as the template for qPCR. qPCR was performed on a CFX96 Real-Time PCR Detection System using CFX Manager Software v3.0 (Bio-Rad, Hercules, CA, USA), with ChamQ Blue Universal SYBR qPCR Master Mix (Vazyme, Nanjing, China). qPCR was performed according to the manufacturer’s instructions. The reaction mixture (20 µL) contained 10 µL of 2 × ChamQ Blue Universal SYBR qPCR Master Mix (Vazyme, Nanjing, China), 0.4 µL of each primer (10 µM), 1 µL cDNA template, and 8.2 µL of ddH_2_O. The qPCR cycling parameters were 95 °C for 30 s, followed by 40 cycles of 95 °C for 10 s and 60 °C for 30 s. Gene-specific primers were designed using Primer-BLAST (v6.0) and synthesized by Tsingke (Changsha, China) (Table 4). Relative gene expression levels were calculated using the 2^−ΔΔCt^ method, normalized to the expression of Elongation factor 1-alpha (EF1A) from *Py. analis*, and presented as mean ± SD (*n* = 4). The data were analyzed and visualized using the GraphPad Prism v8.0.2 software.

## Figures and Tables

**Table 1:** Summary of clean data.

Sample	Total Bases	Clean Reads	Clean Bases	Q20 Rate (%)	Q30 Rate (%)	GC (%)
*Py. analis*	9,278,430,600	61,856,204	9,249,115,462	99.12	96.04	35.97

**Table 2:** Predicted transcript support for venom protein family in *Py. analis*.

Venom Protein Family	Number of Supporting Transcripts
5′-nucleotidase	4
Lysosomal acid lipase	1
Phospholipase A1	3
CRISP	1
Insulin	3
Phospholipase A2	7
Snaclec	1
Phosphatase Acph-1	25
Carboxylesterase-6	44
U8-agatoxin-Ao1a	1
Dipeptidyl peptidase 4	5
Scoloptoxin SSD14	3

**Table 3:** Amino acid sequences of predicted venom-related proteins in *Py. analis*.

Protein Name	Amino Acid Sequence
Crisp2	MISNRKWNHEVARSAQRWAEACMLLTHDDVAGRFIDNYGSCGQNIFVSSHQVPWLFAIKTWWLEKDNFTFGGDNNDLYVIGHYTQLVWATTHQVGCGFTKCDRVGNNKGRAYYSYVCNYCPIGNHPEKLGTPYKRGKPCGMCKRKCHAKKLCTNACPVADYWANCRELHAAWPNWLCDSSTGRGEERTEYCQATCKCKGTIRQ *
PA-IN01	MERVLLFIIFFGTFINLTFEFEMSQKEMKSIKGKFCGSYLAETLSMVCQGMYNHKGKRNMLGNLGLNKVNSREDYEDSENLLHVDEFLPSFPFRPREHARSLIPYDYQKRSRKALIVDECCDKACSFFELSGYCGKP *
PA-IN02	MARFISMLLLILVCLNTAEATDDLLKLLKSRKATRYCGPNLSEVLSLVCRGRYNTPPPVHDIQRRDVSETTFEEEEVDFPYASRAKLFKFIHKLKRRRQKRGVYNECCENPCSYDVLVSYCA *
PA-IN03	MNYMMGHYIAYLVLWTTFTNFSLGAIFTEREMQFMAGKFCGDHLADMLSLVCGGLYNHKGKRTMFSNKIDNNDDYDDTDSLGYLDDSLMAFPFRSKERAKSLINFQYQKRKGNKKGIVDECCHKSCSLLEMRGYCAN *
PLA2PA4	MIEFTSERPYCIFFTDRDFIQHTLLNTEQRKVKITSSEEIDKLEEVCKKRKLQSSYQGGFIYPGTKWCGPGAIADNYTDLGTHRGEDMCCREHDHCPHYIERGECKQGICNKSKFTRSHCDCDATFRRCLQNVNSETANTIGAIFFNVVQIICFKQRNPCSEFQSSERIEENVIEHDKCGIEFAVVERYQPTKTFCCRTKGEKWLH *
SSD14L2	MVDLQNSYSTPSVSSKSVVSRRARCRNSSRRKFDNGMNTRYGEYTKLGVKDHYGYEKRPRCRPLHVVMLLFGILIIAIIIAYFLGAFEKTTQKTTVLVPPNPQKPLPPSSSKLHRFQKAAVCADAPVCAEIGKNILERNGSAVDAAIATLFCNGVVNMQSMGLGGGFLMTIYIRDSKTAVTLDARESAPLNATANMFKNENESKYSGLAVGVPGELRGYWVAHEKYGTLPWKELVQPTIDLCERGYNMTVPQFDVLNKIRNDSNLREWFFDANGKFKPAGSLIKPKKLCDTLKIIADSDGNELYNGTLSKMLLQDIKDMGGIITEEDLQNYTPKWRDPVSVDLNGGDHLYSIPPPGSGILLAFVLNILDGYNFTRDSINNINKTVLTYHRIIEAFKFAYAKRTELGDTDFVNITTLLQNLTSNDYARNIRNKILDNTTHLNPADYGAVFYNREDHGTAHISVLAPNGDAVSVTSTVNIFFGNGVTSESTGIILNNGMDDFSSPNFPNYFELPGSPNNAIAPSKRSLSSMTPTIITDQNGDVRMVIGASGGTKITTSIALVIMRNLWFGDNIKEAVDAPRIHHQLYPMEISYEYGNLQQIVGGLEKLGHNTTRYSDRGSIICALIVQNGTIIANSDYRKGGDVYGI *
Pla1	MKQIISVFFFILFLNSGFGEEEQESKLKNLFTNMHGLVYGKGLMDTITCQEVTPNDFTMLLYTRTSNDTPTHLNISNPQLDTTKNLIILSHGWIQSATTYGMQRIKNEYLKRYDSNIIVVNWEDLAYQLYSTAVCTLPKIGKNIANYLCDLHTKHGLKMENVHMVGHSLGGQLAGHISNDMKQICGQKFGRITGLDPAGPLYVDTPRENRLDSTDATFVQVVHTSGGSFGYYGKCGHADFFVKCGSVQGSCNLQEGQWKMLNELPMPINALSAVVCYHMRALDIWVEAINSDDFKAIPCTYCPIGCPPLLSFGSKKTIMGESCPTDAKGGYYLSTNSESPYAKSSFF *
5-NT	MRIINLVVLIILNLILKNVQSENLKLLILHNNDMHARFEETSRTSGTCKDKSNCYGGFGRVLHVVREARKAAADGTGPPVLYLNAGDTYTGTSWFSVHKWKIAADFMNLLQPDVASLGNHEFDYNVSGILPFLHAVNFPIVAANLDFSKEPELQATNITKSYTIEISGRKIGIIGYLLPESTELANTEDVIFVDEVVAIKEESERLQKEGVNIIIALGHSGYTKDLVIAKEVPLVDLVVGGHSDTFLWNGPQPDLEIPEGPYPTIVKQDSGKEVLVVQPYSYTKYMGRLNVEFDDNGDVVAYSGQPQLLNSLVPQEKDVLDLLEKYRPEVRQLEEEVLGRTKVFLDGTPENCRRKECNLGNLITDAFVAYHSERYEGKYWTDAPIGIYNGGAIRNNIDPVNGTIRGSDLISAFPYNDLVYSMSLSGTDLLHTLEQGVRSNGETSYGEFLQVSGIRYRFDLSKPVGSRIVEAKSRCGSCNTPIYEQINPNKKYRIITREFITNGGDGHTTLKHKGYDKEVEDLNEFEIVSWYIKKMSLILVGEDERVLKEEKSKAYICSQSISLMMFASLFLYNAK *

* Stop codon.

**Table 4:** Primer sequences used for qPCR analysis of putative toxin and reference genes in *Py. analis*.

Accession Number	Gene Name	Primer	Sequence (5′–3′)
TRINITY_DN5639_c0_g1_i1	5′-nucleotidase	Forward	CACGTTGGTTGCACAGGAAG
		Reverse	CGCGACGACGAGACATTTTC
TRINITY_DN19953_c0_g1_i1	Lysosomal acid lipase	Forward	GCTCGACCCGGTTCAAGAAA
		Reverse	CAACAGGTGCCAAAGCTGTC
TRINITY_DN11667_c0_g1_i1	Phospholipase A1	Forward	GGGCATTCATTGGGAGGACA
		Reverse	CCTGGAGATTGCACGAACCT
TRINITY_DN15036_c0_g1_i3	CRISP	Forward	CCCATGATGACGTAGCTGGAA
		Reverse	ACCGACCTGATGGGTTGTTG
TRINITY_DN3672_c0_g1_i5	Insulin	Forward	TGGAAGTAAACCAGCGGCAA
		Reverse	GAAATGCACATGCCCAGCAA
TRINITY_DN11389_c0_g1_i2	Phospholipase A2	Forward	GCAGTCATCGTATCAGGGGG
		Reverse	CAAGCATCTGCGGAATGTCG
TRINITY_DN7940_c0_g1_i1	Snaclec	Forward	ACAGCAGTCAACCGGAAGAG
		Reverse	TACAGTTTCATGTGCGCCCT
TRINITY_DN10483_c0_g2_i1	Phosphatase Acph-1	Forward	TTTGCGCTTTTCCGACATGG
		Reverse	TGGGAATAGACCCGCCAGTA
TRINITY_DN4163_c0_g2_i1	Carboxypeptidase-6	Forward	AAAGGAGTGGATAGCGGCTG
		Reverse	TTCCGATATCCAGGGAGCGA
TRINITY_DN2951_c0_g1_i4	Dipeptidyl peptidase 4	Forward	AACCAATTTTGGCAGCCGTC
		Reverse	AATGTCGGTAAGCCCCTGTC
TRINITY_DN474_c0_g1_i10	Scoloptoxin SSD14	Forward	CTGAGCTTGGTGACACGGAT
		Reverse	GGCATCACCATTCGGTGCTA
TRINITY_DN3142_c0_g1_i1	U8-agatoxin-Ao1a	Forward	CCGCCTACGAAACACTCGAT
		Reverse	ACGTTTTTGTGCGGACTGAAG
TRINITY_DN2512_c0_g1_i1	Elongation factor 1-alpha (EF1A)	Forward	AGGGAGCTAAATTGGAAGGTAAA
		Reverse	GTGGAAGTCGAAGTGCCTTAT

## Data Availability

The original data presented in the study are openly available in Zenodo at https://doi.org/10.5281/zenodo.17646424 (accessed on 23 December 2025).
